# LUZP1 Controls Cell Division, Migration and Invasion Through Regulation of the Actin Cytoskeleton

**DOI:** 10.3389/fcell.2021.624089

**Published:** 2021-04-01

**Authors:** Laura Bozal-Basterra, María Gonzalez-Santamarta, Veronica Muratore, Natalia Martín-Martín, Amaia Ercilla, Jose A. Rodríguez, Arkaitz Carracedo, James D. Sutherland, Rosa Barrio

**Affiliations:** ^1^Center for Cooperative Research in Biosciences (CIC BioGUNE), Basque Research and Technology Alliance, Bizkaia Technology Park, Derio, Spain; ^2^Centro de Investigación Biomédica en Red de Cáncer (CIBERONC), Instituto de Salud Carlos III, Madrid, Spain; ^3^Department of Genetics, Physical Anthropology and Animal Physiology, University of the Basque Country Universidad del País Vasco/Euskal Herriko Unibertsitatea (UPV/EHU), Leioa, Spain; ^4^Ikerbasque, Basque Foundation for Science, Bilbao, Spain; ^5^Biochemistry and Molecular Biology Department, University of the Basque Country (UPV/EHU), Leioa, Spain

**Keywords:** LUZP1, actin cytoskeleton, proliferation, migration, invasion, cancer, centrosome

## Abstract

LUZP1 is a centrosomal and actin cytoskeleton-localizing protein that regulates both ciliogenesis and actin filament bundling. As the cytoskeleton and cilia are implicated in metastasis and tumor suppression, we examined roles for LUZP1 in the context of cancer. Here we show that *LUZP1* exhibits frequent genomic aberrations in cancer, with a predominance of gene deletions. Furthermore, we demonstrate that CRISPR/Cas9-mediated loss of *Luzp1* in mouse fibroblasts promotes cell migration and invasion features, reduces cell viability, and increases cell apoptosis, centriole numbers, and nuclear size while altering the actin cytoskeleton. Loss of *Luzp1* also induced changes to ACTR3 (Actin Related Protein 3, also known as ARP3) and phospho-cofilin ratios, suggesting regulatory roles in actin polymerization, beyond its role in filament bundling. Our results point to an unprecedented role for LUZP1 in the regulation of cancer features through the control of actin cytoskeleton.

## Introduction

The Leucine Zipper Protein 1, LUZP1, was originally identified as a nuclear protein mainly expressed in the brain (Sun et al., 1996; Lee et al., 2001). Recent publications demonstrate that LUZP1 is a centrosome, actin and midbody-localizing protein implicated in ciliogenesis regulation and actin cytoskeleton stability (Wang and Nakamura, 2019a; Bozal-Basterra et al., 2020; Goncalves et al., 2020). In addition to three leucine zipper motifs located at the N-terminus, LUZP1 contains a large number of putative serine/threonine phosphorylation sites (Sun et al., 1996). Mutations in mouse *Luzp1* resulted in cardiovascular defects and cranial Neural Tube Defects (NTD) accompanied by elevated apoptosis of mesenchymal cells, demonstrating its crucial role in embryonic heart and brain development (Hsu et al., 2008). In humans, specific mutations in *LUZP1* have not yet been reported. However, complete deletion of *LUZP1*, as well as other loci, gives rise to 1p36 deletion syndrome that affects approximately 1 in 5,000 newborns (Zaveri et al., 2014). The symptoms include developmental delay, intellectual disability, seizures, vision problems, hearing loss, short stature, distinctive facial features, brain defects, orofacial clefting, congenital heart defects, cardiomyopathy, and renal anomalies. Although the exact contribution of *LUZP1* in the pathogenesis of the 1p36 syndrome is unknown, it has been proposed to contribute to the development of the cardiovascular malformations (Zaveri et al., 2014; Jordan et al., 2015). In addition, a recent study reported that increased *LUZP1* expression in the uterus was associated with higher fibroid risk in humans (Edwards et al., 2019). Furthermore, Poel and colleagues claimed that *LUZP1* downregulation might mediate chemotherapy sensitivity mechanisms in colorectal cancer cells, potentially through cell cycle arrest (Poel et al., 2019). In addition, loss of expression of a LUZP1-interacting tumor suppressor protein named EPLIN (also known as LIM Domain And Actin Binding 1, LIMA1) has been associated with cancer by affecting cancer cell adhesion and migration, and increasing metastatic potential (Jiang et al., 2008; Sanders et al., 2010; Zhang et al., 2011; Liu et al., 2012; Collins et al., 2018). Despite this evidence, focused research on LUZP1 is necessary to elucidate the role that it might have in cellular features underlying cancer development.

LUZP1 has been identified as a new actin-associated protein, through interactions with ACTR2 (Actin Related Protein 2, also known as ARP2) (Hein et al., 2015) and filamin A (FLNA), with a likely role in bundling of actin filaments (Wang and Nakamura, 2019a,b; Bozal-Basterra et al., 2020; Goncalves et al., 2020). LUZP1 shows homology to FILIP1 (Filamin A Interacting Protein 1), a protein interactor of filamin and actin (Nagano et al., 2004; Gad et al., 2012), and FILIP1L (FILIP1 Like), a suppressor of tumor cell migration (Kwon et al., 2014). Actin cross-linking factors play a role in coordination of migration and proliferation.

Actin is one of the most abundant proteins in cells and plays crucial roles in cytokinesis during cell division, protrusion of the leading edge of motile cells and maintaining the physical integrity of the cell (Pollard and Cooper, 2009). The organization of filamentous actin (F-actin) network and the formation of cell–matrix adhesions in response to extracellular stimuli are controlled by small GTPases of the Rho family (Etienne-Manneville and Hall, 2002). In their activated GTP-bound state, Rho GTPases can regulate multiple downstream effector pathways. Both Rac1 (Rac Family Small GTPase 1) and RhoA (Ras Homolog Family Member A) GTPases have been reported to activate a pathway that results in the inhibition of cofilin through serine 3 phosphorylation. Rac1 is mostly linked to lamellipodia extension and the formation of nascent adhesions, whereas RhoA stimulates stress fibers formation and maturation of cell–matrix adhesions (Rottner et al., 1999). The activation of the WAVE (WASP (Wiskott-Aldrich syndrome protein)-family verprolin homology protein) and ARP2/3 (Actin Related Protein 2/3 Complex Subunit 2/3) complexes downstream of Rac1 initiates actin polymerization (Eden et al., 2002).

In this work, we demonstrate that heterozygous loss of *LUZP1* is frequent in different cancer types. *Luzp1*-depleted cells exhibit defects in migration/invasion and cell viability, with larger nuclei and multiple centrioles. These differences may contribute to increased apoptosis observed in *Luzp1*-knockout cells. Our findings uncover a functional relationship between *Luzp1* and characteristic features of tumors via regulation of the actin cytoskeleton. These results are particularly relevant, as they may shed light on the molecular mechanisms of cancer.

## Materials and Methods

### Cell Culture

Mouse Shh-LIGHT2 cells (kind gift of A. McGee, Imperial College) (Taipale et al., 2000), Luzp1^–/–^ cells, + LUZP1 (Bozal-Basterra et al., 2020) and human HEK 293FT (Invitrogen), were cultured at 37°C and 5% CO_2_ in Dulbecco’s modified Eagle medium (DMEM) supplemented with 10% fetal bovine serum (FBS, Gibco) and 1% penicillin/streptomycin (Gibco).

### CRISPR-Cas9 Genome Editing

HEK 293FT cell *LUZP1* locus was targeted by CRISPR-Cas9 to generate 293^LUZP1KO^ cells. Two high-scoring sgRNAs were selected^1^ to target near the initiation codon (sg2: 5′-CTTAAATCGCAGGTGGCGGT_TGG-3′; sg3: 5′-CTTCAA TCTTCAGTACCCGC_TGG-3′). These sequences were cloned into px459 2.0 (Addgene #62988; kind gift of F. Zhang, MIT), for expressing both sgRNAs and Cas9 with puromycin selection. Transfections were performed in HEK 293FT cells with Lipofectamine 3000 (Thermo). Twenty-four hours after transfection, transient puromycin selection (0.5 μg/ml) was applied for 48 h to enrich for transfected cells. Cells were plated at clonal density, and well-isolated clones were picked and propagated individually. Loss-of-function mutations were confirmed by PCR-sequencing (Bozal-Basterra et al., 2020).

### Western Blot Analysis

Cells were lysed in cold RIPA buffer (Cell Signaling Technology) supplemented with 1x protease inhibitor cocktail (Roche), and also in some cases with PhosphoStop 1x (Roche). Lysates were kept on ice for 30 min vortexing every 5 min and then cleared by centrifugation (25,000 × g, 20 min, 4°C). Supernatants were collected and protein content was quantified by BCA protein quantification assay (Pierce). After SDS-PAGE and transfer to nitrocellulose membranes, blocking in 5% milk, or in 5% BSA (Bovine Serum Albumin, Fraction V, Sigma) in PBT (1x PBS, 0.1% Tween-20) was performed. In general, primary antibodies were incubated overnight at 4°C and secondary antibodies for 1 h at room temperature (RT). Antibodies used: anti-vinculin (Sigma, 1:1,000), anti-cofilin and anti-phospho-cofilin (Cell Signaling Technology, 1:1,000), anti-Rac1 and anti-pRac1 (Cell Signaling Technology, 1:1,000), [anti-Actr3 (Machesky et al., 1997), 1:1,000], anti-GAPDH (Glyceraldehyde-3-Phosphate Dehydrogenase; Proteintech, 1:1,000) and anti-actin (Sigma, 1:1,000). Secondary antibodies were anti-mouse or anti-rabbit HRP-conjugates (Jackson Immunoresearch). Proteins were detected using Clarity ECL (BioRad) or Super Signal West Femto (Pierce). Quantification of bands was performed using ImageJ software and normalized against GAPDH or actin levels. At least three independent blots were quantified per experiment.

### Immunostaining

Shh-LIGHT2 cells and HEK 293FT cells were seeded on 11 mm coverslips (15,000–25,000 cells per well; 24-well plate). After washing once with cold 1x PBS, cells were fixed with methanol 100% for 10 min at −20°C or with 4% PFA supplemented with 0.1% Triton X-100 in PBS for 15 min at RT. Then, coverslips were washed 3 times with 1x PBS. Blocking was performed for 1 h at 37°C in blocking buffer (2% fetal calf serum, 1% BSA in 1x PBS). Primary antibodies were incubated overnight at 4°C and cells were washed with 1x PBS 3 times. We used antibodies anti-gamma-tubulin (Proteintech, 1:160), rabbit anti-Cleaved Caspase-3 (Cell Signaling Technology 9661S, 1:200) and anti-vinculin (Sigma hVIN-1, 1:200).

Donkey anti-mouse or anti-rabbit secondary antibodies (Jackson Immunoresearch) conjugated to Alexa 488 or Alexa 568 (1:200) and Alexa 568-conjugated phalloidin (Invitrogen 1:500), were incubated for 1 h at 37°C, followed by nuclear staining with DAPI (10 min, 300 ng/ml in PBS; Sigma). Fluorescence imaging was performed using an upright fluorescent microscope (Axioimager D1, Zeiss).

### Cell Cycle Analysis by Quantitative Image-Based Cytometry

Cells were seeded in 96-well plates to be approximately 80% confluent the day of the EdU (5-ethynyl-2 deoxyuridine) labeling. Cell cultures were incubated 30 min with 10 μM EdU (Sigma, #900584) at 37°C in their own culture medium. Cells were washed and then fixed 15 min in 4% (w/v) formaldehyde solution in phosphate buffered saline (PBS). Following 3x PBS washes, fixed cells were permeabilized with 0.2% Triton X-100 in PBS for 30 min at room temperature. After washing with PBS, cells were directly incubated 30 min at room temperature (RT) in click reaction buffer. For 1 ml of click reaction buffer, following amounts of the different components are mixed in 680 μl milliQ water: 100 μl 1M Tris-HCl pH = 8; 20 μl 100 mM CuSO4; 0.5 μl Alexa Fluor^TM^ 647 Azide 1 μg/μl (AA648; Invitrogen) and 200 μl 0.5 M Ascorbic Acid. Finally, cells were washed 3x with PBS and incubated in 0.5 μg/ml DAPI-containing PBS for at least 30 minutes or until imaging.

For quantitative image-based cytometry images (QIBC), EdU labeled cells were obtained in an automated manner with the ScanR acquisition software controlling a motorized Olympus IX-83 wide-field microscope. Images from 3 technical replicates in 8 independent experiments were then processed using the ScanR image analysis software and analyzed with TIBCO Spotfire software.

### Fluorescence-Activated Cell Sorting

To evaluate apoptosis, Shh-LIGHT2 cells were washed with 1x PBS and then stained with Annexin V (BD Biosciences) and DRAQ7 (Biostatus Ltd.). Data from 4 biological replicates were collected on a Fluorescence-activated Cell Sorting (FACS) Canto (BD Biosciences).

### Cell Viability Assay

5 × 10^3^ cells were plated in triplicate in 12-well plates. Twenty-four hours later, the cells were considered day 0 (t_0_) and were fixed in formalin 10% for 15 min. The same procedure was performed after 3 and 6 days. Cell viability was measured by staining with crystal violet (0.1% in 20% methanol) for 45 min at RT. After washing 3 times with water, all samples were air dried. The precipitate was solubilized in 10% acetic acid for 20 min at RT, and the absorbance was measured at 595 nm. For each timepoint, 4 biological replicates were measured.

### Wound-Healing Assay

Shh-LIGHT2 control, Luzp1^–/–^ mutant cells and + LUZP1 cells were grown in 24-well plates and a scratch wound was performed using a 20 μl pipette tip. Subsequently, medium was changed to remove detached cells. Pictures were taken at three different positions per sample in three technical replicates and at least eight biological replicates were analyzed of each. The scratch width was measured using ImageJ Fiji.

### Filopodia Quantification

Filopodia were quantified by staining cells with Alexa 594-conjugated wheat germ agglutinin (WGA) and using FiloQuant, a plugin for the ImageJ software (Figure 2F and Supplementary Figure S1A; Jacquemet et al., 2017). The average number of filopodia of individual cells in 7 biological replicates was pooled together to perform statistical analysis.

### Three-Dimensional Spheroid Invasion Assay

Both WT and Luzp1^–/–^ Shh-LIGHT2 cells were suspended in DMEM medium plus 5% Methyl cellulose (Sigma) at 14,0000 cells/ml. Cell spheroids were subsequently formed by serial pipetting of 25 μl into the lid of a 10 cm dish (700 cells/spheroid) and incubated in an inverted position. After 48 h, cell spheroids were embedded into a volume of 300 μl of 2.3 mg/ml bovine collagen type I matrix (Advanced) and transferred to individual wells of a 24-well plate. Four hours later, each well was filled with complete media. Collective cell invasion was monitored using a Nikon Eclipse TS100 Live Imaging microscope. Images were taken just after adding complete medium to the collagen-embedded cells (*t* = 0 h) and 18 h later (*t* = 18 h). The area of each individual spheroid was measured in 3 technical replicates and in at least 3 biological replicates using ImageJ analysis program. The fold change in invasive area was determined by dividing the final area (at 18 h) by the initial area (at 0 h) in each cell type.

### Transwell Assay

For transwell assay (24-well format), 2 × 10^4^ WT, Luzp1^–/–^ and + LUZP1 Shh-LIGHT2 cells were seeded in the upper chamber in serum-free medium (0.5 ml; inserts 6.5 mm, 8 μm pore size; Corning Costar). The lower chamber was loaded with 1 ml medium supplemented with 10% FBS. After 6 h of incubation at 37°C with 5% CO_2_, the migrated cells in the membrane were stained by DAPI. Images were obtained in an automated manner with the ScanR acquisition software controlling a motorized Olympus IX-83 wide-field microscope. Images from 6 independent experiments were then processed using the ScanR image analysis software.

### Bioinformatics Analysis

Patient copy number and mRNA expression information was obtained from cBioPortal (Cerami et al., 2012; Gao et al., 2013), Cancertool (Cortazar et al., 2018), and TCGA Copy Number Portal^2^.

### Statistical Analysis

Statistical analysis was performed using GraphPad 6.0 software. Data were analyzed by Shapiro-Wilk normality test and Levene’s test of variance. We used two tailed unpaired Student’s *t*-test or Mann Whitney-*U* tests for comparing two groups, One-way ANOVA or Kruskall-Wallis and the corresponding *post hoc* tests for more than two groups and two-way ANOVA to compare more than one variable in more than two groups. *P-*values were represented by asterisks as follows: ^∗^*P* < 0.05; ^∗∗^*P* < 0.01; ^∗∗∗^*P* < 0.001; ^****^P < 0.0001. Differences were considered significant when *P* < 0.05.

## Results

### Copy Number Alterations and Aberrant *LUZP1* Gene Expression Are Common Events in Cancer

**FIGURE 1:**
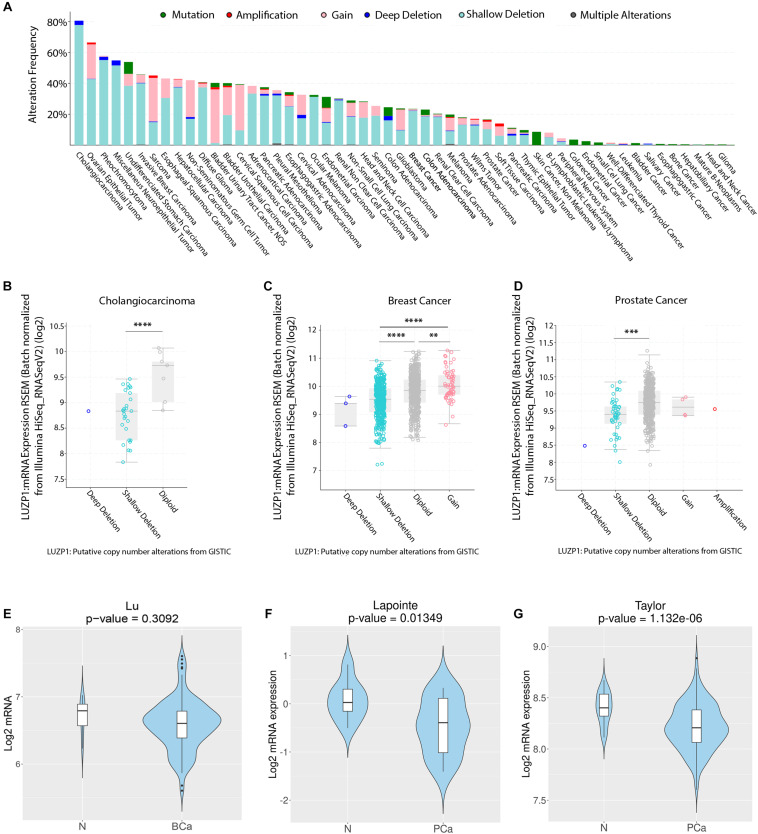
Copy number alterations and aberrant *LUZP1* gene expression in cancer. **(A)** Copy number alterations (CNA) (mutations-green; amplifications-red; gain-light pink; deep deletions-blue; shallow deletions-aquamarine; multiple alterations-gray) in different TCGA tumor types (*n* = 10,967). **(B–D)** *LUZP1* mRNA expression (RNAseq) sorted by CNA in **(B)** Cholangiocarcinoma (*n* = 195), **(C)** Breast (*n* = 1,918), and **(D)** Prostate Cancer samples (*n* = 324). When *n* > 3, data were analyzed by One-way ANOVA or Kruskall-Wallis and the corresponding *post hoc* test. **(E)** Violin plots depicting the expression of LUZP1 between non-tumoral (N) and breast cancer specimens (BCa) in the Lu dataset (Lu et al., 2008). **(F,G)** Violin plots depicting the expression of LUZP1 between non-tumoral (N) and prostate cancer specimens (PCa) in Lapointe et al. (2004) **(F)** and Taylor et al. (2010) **(G)** datasets. The Y-axis represents the Log2-normalized gene expression (fluorescence intensity values for microarray data or sequencing read values obtained after gene quantification with RSEM and normalization using Upper Quartile in case of RNAseq). Student *T*-test was performed in order to compare the mean gene expression between two groups. ^∗∗^*P* < 0.01; ^∗∗∗^*P* < 0.001; ^****^P < 0.0001.

The process of cellular transformation from normal to malignant cellular behavior derives from the acquisition of genomic aberrations and the development of cancer hallmarks, such as activation of invasive and migratory phenotypes and resistance to apoptosis (Hanahan and Weinberg, 2011). We first aimed to characterize the genomic alterations of *LUZP1* reported in various cancer types using a publicly available webtool (cBioPortal) (Cerami et al., 2012; Gao et al., 2013). Interestingly, we found genomic aberrations in *LUZP1* at high frequency in cancer specimens, reaching an alteration frequency of almost 80% in cholangiocarcinoma and greater than 40% in 13 out of the 54 cancer types that we analyzed (Figure 1A). Importantly, shallow deletions (most possibly heterozygous deletions according to cBioPortal) were the most prevalent copy number alterations event in a large fraction of cancers (Figure 1A, aquamarine). We next evaluated whether copy number aberrations would influence *LUZP1* gene expression across several cancer types based on the TGCA Pancancer Atlas database (cBioPortal). As predicted, shallow and deep deletions (most probably homozygous deletions, according to cBioPortal) were associated to lower *LUZP1* mRNA expression compared to diploid, gain or amplification events in cholangiocarcinoma (Figure 1B), breast cancer (Figure 1C) and prostate cancer (Figure 1D), indicating that genomic events are, at least in part, responsible for reduced LUZP1 expression associated to cancer. In line with the genomic analysis and the gene dosage-mRNA expression association, using Cancertool (Cortazar et al., 2018) we found the mRNA levels of *LUZP1* to be significantly reduced in prostate cancer (Figures 1F,G), and non-significantly in breast cancer (Figure 1E). From our analysis, *LUZP1* emerges as a potential cancer-relevant gene, exhibiting genomic and transcriptional perturbation pattern suggestive of a tumor suppressive function.

**FIGURE 2:**
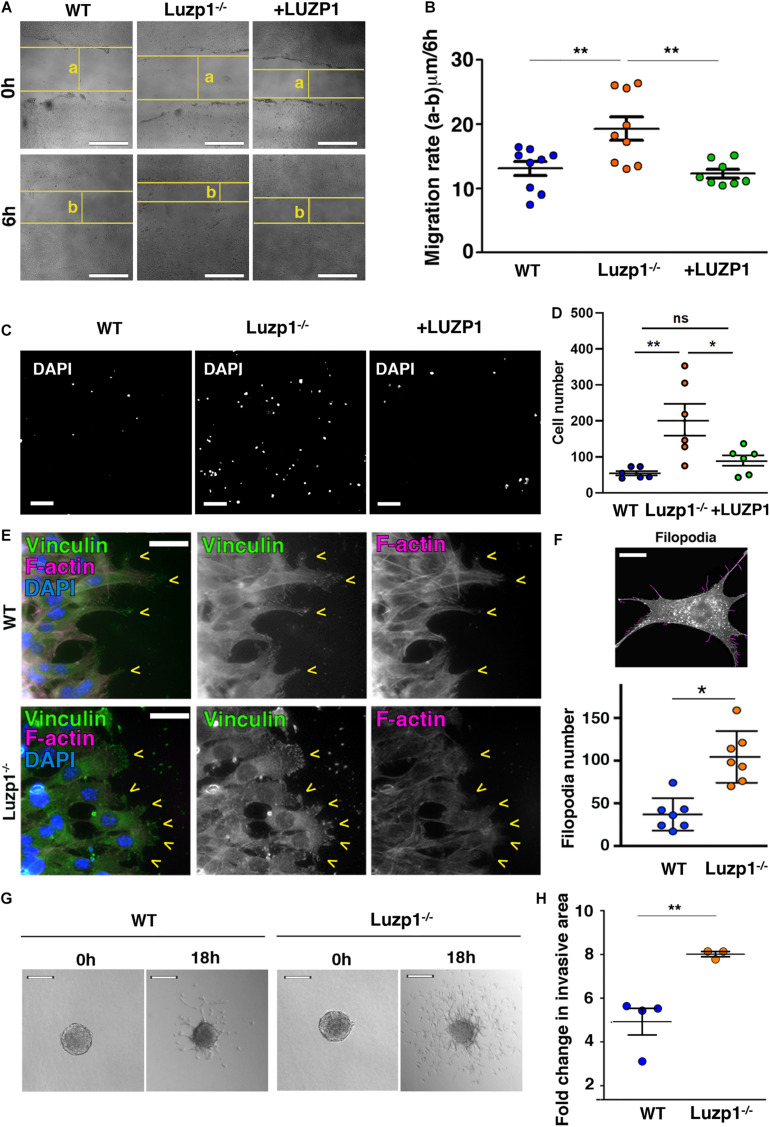
Luzp1^–/–^ cells exhibit cell migration and invasion defects. **(A)** Representative bright-field micrographs of the wound healing assays performed on Shh-LIGHT2 WT, Luzp1^–/–^ and Luzp1^–/–^ cells rescued with *Luzp1-YFP* (+ LUZP1). The horizontal yellow lines represent the wound boundary; “a” and “b” are the distances between wound boundaries just after wound was made (0 h) and 6 h later (6 h), respectively. Scale bar, 200 μm. **(B)** Quantification of wound healing in **(A)** calculated by subtracting distance “b” to distance “a” and dividing the result by 6 h (*n* > 8). Data were analyzed by One-way ANOVA and Bonferroni *post hoc* test. **(C)** Representative images of migrating cells of a transwell assay are shown. Cells were detected by DAPI (white). Scale bar, 100 μm. **(D)** Graph representing the number of migrating cells. **(E)** Micrographs of WT and Luzp1^–/–^ cells during wound healing assay. Focal adhesions were detected by anti-vinculin antibody (green), F-actin by phalloidin (magenta) and nuclei by DAPI (blue). Yellow arrowheads point at lamellipodia. Black and white images show the single green and magenta channels. Scale bar, 10 μm. **(F)** Upper panel: example of filopodia detection by Filoquant plugin for ImageJ (in magenta) from an original picture of a Luzp1^–/–^ cell (in black and white). Scale bar, 5 μm. Lower panel: graphical representation of filopodia number of WT (*n* = 7; blue dots) and Luzp1^–/–^ cells (*n* = 7; orange dots). Imaging was performed using widefield fluorescence microscopy (Zeiss Axioimager D1, 63x objective). *P*-value was calculated using Mann Whitney test. **(G)** Invasive growth of WT or Luzp1^–/–^ cells was analyzed in 3D collagen matrix. Images were taken at *t* = 0 h and *t* = 18 h. Representative images are shown. Scale bar, 25 μm. **(H)** Representation of the fold change in invasion resulting of dividing the area covered by the cells at *t* = 18 h by the area covered by the cells at *t* = 0 h. Data were analyzed by ANOVA and Bonferroni *post hoc* test or Student’s *t*-test. The graphs in **(B,D,F)** represent the Mean and SEM. Data were analyzed by ANOVA and Bonferroni *post hoc* test or Student’s *t*-test. ^∗^*P* < 0.05; ^∗∗^*P* < 0.01.

### *Luzp1* Deletion Increases Cell Migration and Invasion

Based on the genetic alterations of *LUZP1* in cancer specimens as mentioned above, we analyzed intrinsic cellular features altered in cancer, such as cell migration and invasion *in vitro*, to see how they might be affected with loss of *Luzp1*. Using CRISPR/Cas9 gene editing directed to exon 1 of murine *Luzp1*, we previously generated Shh-LIGHT2 mouse embryonic fibroblasts (Taipale et al., 2000) null for *Luzp1* (Luzp1^–/–^ cells), and additionally rescued the same cells by expression of human *LUZP1-YFP* fusion (+ LUZP1 cells) (Bozal-Basterra et al., 2020). Interestingly, Luzp1^–/–^ cells elicited a remarkable increase in migratory capacity compared to WT cells, as shown by wound healing assays (Figures 2A,B). This phenotype was suppressed in + LUZP1 rescue cells. We further confirmed the heightened migratory capacity of Luzp1^–/–^ cells by Boyden chamber or transwell assay (Figures 2C,D).

To migrate, a cell must coordinate a number of different inputs into appropriate cellular responses. Vinculin and phalloidin staining was performed in migrating cells to examine focal adhesions and visualize cell shape, respectively. We observed that Luzp1^–/–^ cells form more lamellipodia than WT at the leading edge of migrating cells (Figure 2E). Filopodia and filopodia-like protrusions are prominent features of migrating cells *in vitro* (Petrie and Yamada, 2012; Jacquemet et al., 2013; Paul et al., 2015). In concordance with increased migration, Luzp1^–/–^ cells displayed more filopodia than WT cells (Figure 2F and Supplementary Figure S1A). To further characterize the regulation of invasive properties by LUZP1, we generated spheroids to measure the invasive growth into 3D collagen matrix. The results show that the Luzp1^–/–^ cells showed higher invasive capacity than WT cells (Figures 2G,H). Taken together, these data revealed that *Luzp1* loss leads to an increase in cell migration and invasion.

### *Luzp1* Loss Reduces Cell Viability, Alters Cell Cycle and Increases Apoptosis

**FIGURE 3:**
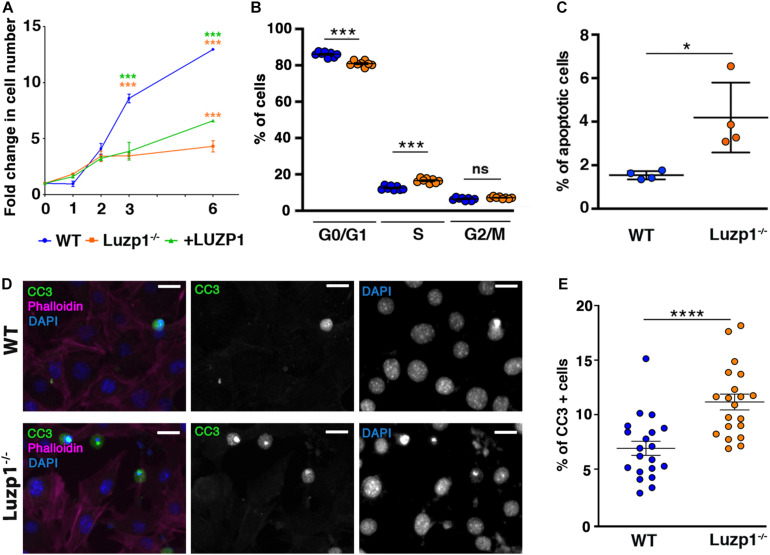
Luzp1^–/–^ cells exhibit lower viability and higher apoptosis than WT. **(A)** Graphical representation of fold change in cell numbers of WT (*n* = 5; blue line), Luzp1^–/–^ (*n* = 5; orange line) and + LUZP1 cells (*n* = 5; green line). *P*-values were calculated using Two-way ANOVA and Sidak’s multiple comparisons test. **(B)** Graphical representation of the percentage of WT (blue dots) and Luzp1^–/–^ cells (orange dots) in G0/G1, S or G2/M phases analyzed by EdU labeling and QIBC. *P*-values were calculated using Two-way ANOVA. **(C)** Graphical representation of the results of FACS analysis in Supplementary Figure S1B using Annexin V and DRAQ7 staining to determine the percentage of apoptotic cells in WT and Luzp1^–/–^ cells in (*n* = 4). *P*-value was calculated using Mann Whitney-*U* test. **(D)** Representative micrographs showing WT and Luzp1^–/–^ cells stained with the marker of mid-stage apoptosis cleaved caspase-3 (CC3) (green), phalloidin (magenta), and DAPI (blue). Black and white images show the single green and blue channels. Images were detected using Zeiss fluorescence microscope (Axio Imager D1), × 40 objective. Scale bar: 25 μm. **(E)** Graphical representation of quantification of CC3-positive cells in **(D)** showing the percentage of apoptotic cells in WT and Luzp1^–/–^ cells (*n* = 20 micrographs, objective 20x). *P*-value was calculated using Mann Whitney-*U* test. The graphs in **(A–C,E)** represent the Mean and SEM. ^∗^*P* < 0.05; ^∗∗∗^*P* < 0.001; ^****^P < 0.0001.

An increase in cell migration and invasion could be influenced by a differential rate of cell proliferation in Luzp1^–/–^ cells. To check this, we analyzed cell viability in WT, Luzp1^–/–^ and + LUZP1 cells using crystal violet assay. Surprisingly, Luzp1^–/–^ cells exhibited a significant reduction in cell numbers at day 3 and 6 after seeding compared to WT cells (Figure 3A). + LUZP1 cells partially rescued the cell viability impairment (Figure 3A, green line). In addition, EdU labeling and QIBC analysis revealed significant changes in cell cycle (Figure 3B), showing fewer Luzp1^–/–^ cells in G0/G1 phases and more Luzp1^–/–^ cells in S phase compared to WT. To determine the level of apoptosis occurring within each population, we performed FACS analysis of cells co-stained for Annexin V and DRAQ7. We detected more apoptotic cells among the Luzp1^–/–^ cell population (Figure 3C and Supplementary Figure S1B). In agreement with these results, we performed immunofluorescence staining for cleaved caspase-3 (CC3), a marker of mid-stage apoptosis. More apoptotic cells were detected among the Luzp1^–/–^ cell population compared to WT cells (Figures 3D,E). Our results showed a global reduction in cell viability rate. The overall increase in proliferation in Luzp1^–/–^ cells by EdU labeling might be counterbalanced by an increase in apoptosis in Luzp1^–/–^ cells compared to WT. Whether or not these phenotypes are independently linked to LUZP1 function, or one is a consequence of the other, remains to be determined.

### *Luzp1* Depletion Affects Nuclear Size and Centriole Number

**FIGURE 4:**
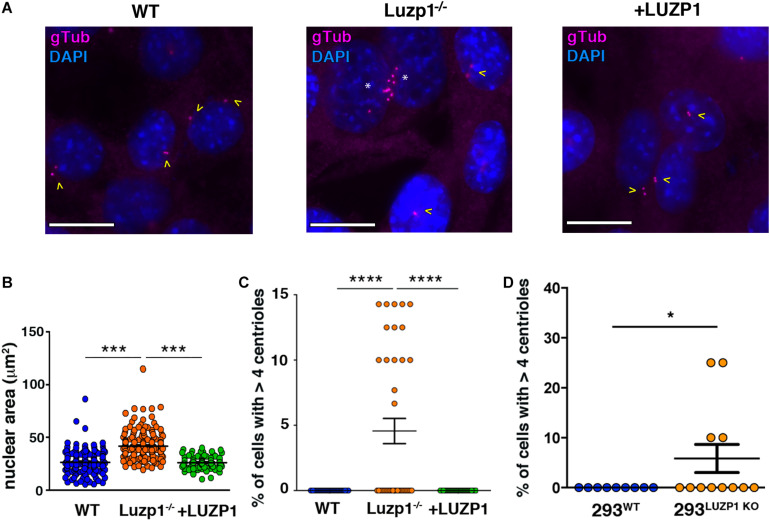
Luzp1^–/–^ cells exhibit bigger nuclei and multiple centrioles. **(A)** Micrographs showing centrioles in wild-type Shh-LIGHT2 cells (WT), Shh-LIGHT2 cells lacking *Luzp1* (Luzp1^–/–^) and Luzp1^–/–^ cells rescued with *Luzp1-YFP* (+ LUZP1) analyzed during cycling conditions. Centrioles were visualized using gamma-tubulin antibody (gTub, magenta) and nuclei were counterstained using DAPI (blue). Scale bar, 5 μm. Cells containing 2 centrioles are marked with yellow arrowheads and those containing multiple centrioles with white asterisks. **(B)** Graphical representation of nuclear area of Shh-LIGHT2 WT (*n* = 178 micrographs; blue dots), Luzp1^–/–^ (*n* = 185 micrographs; orange dots) and + LUZP1 cells (*n* = 90 micrographs; green dots). *P*-values were calculated using Kruskall-Wallis and Dunn’s multiple comparisons tests. **(C)** Graphical representation of the percentage of cells that exhibit more than 4 centrioles in **(A)**. WT, *n* = 37 micrographs, blue dots; Luzp1^–/–^, *n* = 38 micrographs, orange dots; + LUZP1, *n* = 35 micrographs, green dots. *P*-values were calculated using Kruskall-Wallis and Dunn’s multiple comparisons tests. **(D)** Graphical representation of the percentage of cells that exhibit more than 4 centrioles in wild-type HEK 293FT (293^WT^) and HEK 293FT cells lacking *Luzp1* (293^LUZP1KO^). 293^WT^, *n* = 9 micrographs, blue dots; 293^LUZP1KO^, *n* = 12 micrographs, orange dots. *P*-values were calculated using Mann Whitney-*U* test. The graphs in **(B–D)** represent the Mean and SEM. ^∗^*P* < 0.05; ^∗∗∗^*P* < 0.001; ^****^P < 0.0001.

The regulation of cell division, migration and invasion is influenced by the size and number of intracellular structures (Ogden et al., 2013; Bell and Lammerding, 2016). We noted a striking alteration in nuclear size, which was significantly increased in Luzp1^–/–^ cells and suppressed in + LUZP1 rescue cells (Figures 4A,B). As LUZP1 localizes to centrosomes and plays a role in ciliogenesis, (Bozal-Basterra et al., 2020; Goncalves et al., 2020), we examined centrosomes from Luzp1^–/–^ cells throughout the cell cycle and noticed an additional phenotype: *Luzp1* depletion resulted in the heightened incidence of cells with more than four centrioles (Figure 4C). This phenotype was suppressed in + LUZP1 rescue cells. The presence of multiple centrioles was further verified in 293^LUZP1–KO^ cells (Figure 4D). Taken together, these results point to a regulatory role for LUZP1 in centrosome duplication, cytokinesis, or both, resulting in larger nuclei and multiple centrioles.

### Potential Role for *LUZP1* in Regulation of Actin Polymerization

**FIGURE 5:**
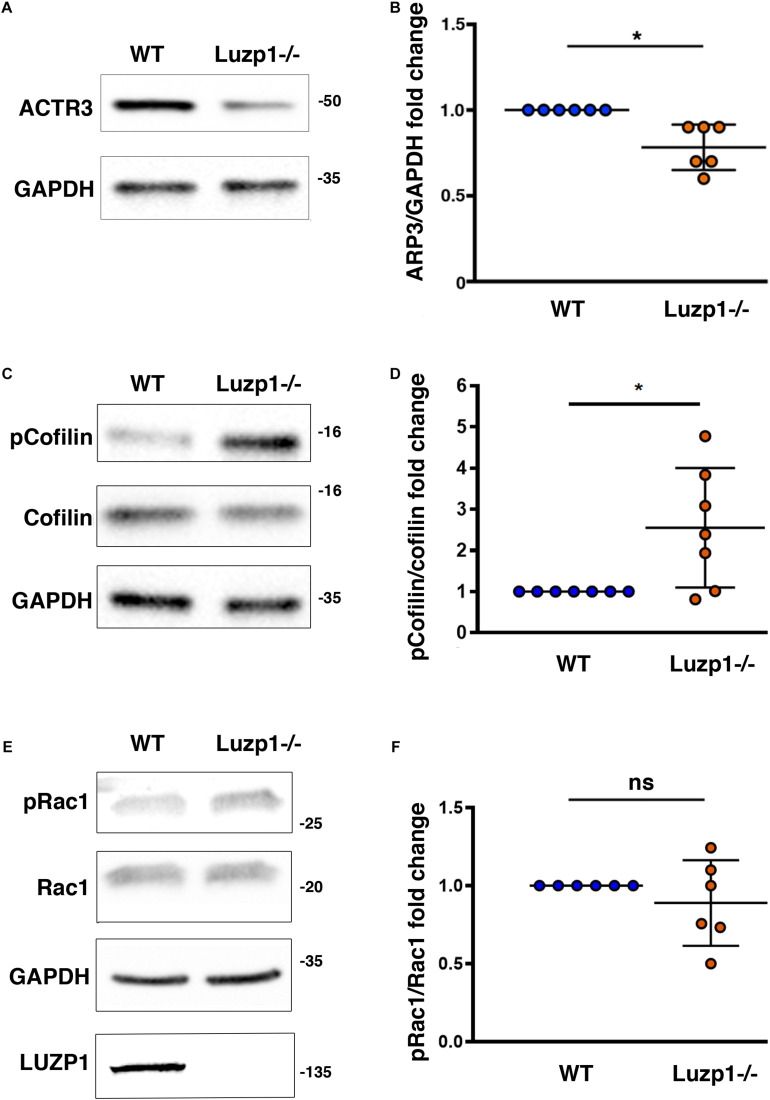
Changes in levels of proteins related to actin polymerization in Luzp1^–/–^ cells. **(A,C,E)** Representative western blot of total lysates of Shh-LIGHT2 WT and Luzp1^–/–^ cells. Note a reduction in ACTR3 (**A**) and an increase in phosphorylated Cofilin (p-Cofilin):Cofilin ratio (**C**) in Luzp1^–/–^ cells. No significant changes were observed in phosphorylated Rac1 (pRac1) **(E)**. Specific antibodies against ACTR3, Cofilin, phospho-Cofilin, pRac1, Rac1 actin and GAPDH were used. Molecular weight markers (kDa) are shown to the right. **(B,D,F)** Graphical representation of the fold change of ACTR3/GAPDH ratios obtained in (**A**), the p-Cofilin/Cofilin ratios obtained in **(C)** and the pRac1/Rac1 ratios obtained in **(E)**. Data from at least three independent experiments pooled together are shown. *P*-values were calculated using two-tailed unpaired Student’s *t*-test. ns: no significant. The graphs in **(B,D,F)** represent the Mean and SEM. ^∗^*P* < 0.05.

Based on the association of LUZP1 to F-actin and the reduction of actin filaments that we had previously observed in Luzp1^–/–^ cells (Bozal-Basterra et al., 2020), as well as the reported interaction of LUZP1 with Arp2 (Hein et al., 2015; Goncalves et al., 2020), we wondered whether other regulators of actin polymerization might be altered. Using western blot, we observed a decrease in total ACTR3 levels (Figure 5A,B), that was accompanied by an increase in phosphorylated cofilin in Luzp1^–/–^ cells (Figures 5C,D). Activation of Rac1 GTPase activity leads to changes in actin polymerization mediated by cofilin and the ARP2/3 complex, but in terms of Rac1 activation by phosphorylation (pRAC1:RAC1 ratio), we did not find significant differences between Luzp1^–/–^ and WT cells (Figures 5E,F). Taken together, these data point to multiple roles for Luzp1 in actin cytoskeleton dynamics.

## Discussion

Our results support that *Luzp1* depletion promotes cell migration and invasion, potentially through regulation of the actin cytoskeleton. Importantly, these features are not ascribed to elevated cell viability, since the lack of *Luzp1* leads to a reduction in cell numbers and additionally, an increase in cell apoptosis. Our results coincide with the reported anti-proliferative effect of *Luzp1* downregulation in colorectal cancer cells (Poel et al., 2019) and the reported increase in proliferation due to *Luzp1* upregulation in uterine fibroids (Edwards et al., 2019). Considering the interaction between LUZP1 and LIMA1/EPLIN, our results mirror those showing increased metastatic potential upon loss or downregulation of the tumor suppressor EPLIN (Jiang et al., 2008; Sanders et al., 2010; Zhang et al., 2011; Liu et al., 2012; Collins et al., 2018; Goncalves et al., 2020). In fact, we cannot rule out the possibility that LUZP1 and EPLIN (and/or other LUZP1 interactors) might have a cooperative role in the context of cancer. These findings, combined with the fact that *LUZP1* is frequently deleted in many human cancer types, support the hypothesis that LUZP1 has tumor suppressor potential in certain cancers.

### LUZP1 Affects Cell Migration and Invasion

Although actin stress fibers contribute to cell shape and adhesion, their exact role in cell migration/invasion has been debated. Stress fibers are absent from several highly motile cells, such as leukocytes (Valerius et al., 1981) and amoeba of *Dictyostelium discoideum* (Rubino et al., 1984). These observations, together with the relative lack of stress fibers in cells grown in three-dimensional matrices have led to the suggestion that they are not essential for cell migration (Burridge et al., 1988). Indeed, it is possible that, under certain conditions, stress fibers might inhibit motility (Badley et al., 1980; Herman et al., 1981; Cramer et al., 1997; Kemp and Brieher, 2018). These studies match our observations that Luzp1^–/–^ cells, which contain less stress fibers than WT cells (Bozal-Basterra et al., 2020), are more motile than WT.

Many cellular proteins are involved in the tight regulation of actin assembly, which directly influences cell migration and invasion. Actin-related proteins act synergistically to maintain a pool of unpolymerized actin monomers, nucleate, elongate and cap actin filaments, promote dissociation of Pi from ADP-Pi-subunits, sever actin filaments, and crosslink filaments into higher order structures. The Rho GTPases have been studied in association with their roles in the regulation of cell division, migration and invasion, mainly via actin filament organization (Aspenstrom et al., 2004). For instance, RhoD (Ras Homolog Family Member D) binds FILIP1, which binds FLNA and has a crucial function in cell migration in the brain (Nagano et al., 2004; Gad et al., 2012). Interestingly, the domain of FILIP1 that binds RhoD (nucleotides 431–778) has more than 40% homology with the equivalent region of LUZP1. Furthermore, a third related protein, FILIP1L, also inhibits tumor cell migration and invasion in colorectal cancer models (Park et al., 2016). Whether the roles of LUZP1, FILIP1, and FILIP1L are distinct or redundant remains undefined. Future mechanistic studies on how LUZP1 and related proteins regulate actin dynamics will be necessary to understand their roles in these diverse actin-driven cellular processes.

### LUZP1 Has a Role in Cell Division

During mitosis the actin cytoskeleton must rearrange and localize to the contractile ring during cytokinesis (Heng and Koh, 2010). This recruitment of F-actin and actin regulatory proteins to the cell cortex during mitosis is essential for the interaction between astral microtubules and cortical actin, which is believed to be important in regulating mitotic spindle orientation (Rankin and Wordeman, 2010). The role of actin and its regulatory proteins in these processes ranges from regulating centrosome separation to proper spindle assembly and orientation and to elongate kinetochore microtubules (Firat-Karalar and Welch, 2011). We observed that Luzp1^–/–^ cells exhibited multiple centrioles, bigger nuclei, decreased cell viability and increased apoptosis. The increased apoptosis seen in our study matches the elevated apoptosis reported in the neuroepithelium of the NTD *Luzp1* KO mouse hindbrain, which displays NTD (Hsu et al., 2008). Whether some or all of these phenotypes are due to the reduced actin cytoskeleton seen in Luzp1^–/–^ cells, or due to actin-independent roles for LUZP1, remains to be determined.

Moreover, it was previously found that LUZP1 localizes not only to centrioles and actin cytoskeleton, but also to the midbody in dividing cells (Bozal-Basterra et al., 2020; Goncalves et al., 2020). The midbody is formed at the intercellular bridge in the last phase of cytokinesis and contains crucial proteins for the abscission between the dividing cells (D’Avino and Capalbo, 2016). While one explanation for the multiple centrioles in Luzp1^–/–^ cells could be failed cytokinesis, we did not observe an increase in cells with multiple nuclei, and QIBC analysis did not reveal increased polyploidy in cells, even though nuclei were increased in size. The timing of abscission can influence karyoplasmic ratios, which are thought to be linked to metastatic properties (Rizzotto and Schirmer, 2017). Understanding the role of LUZP1 in the midbody, namely in timing and dynamics of contractile ring formation and contraction, as well as abscission, would be of major interest.

### LUZP1 May Affect Both Polymerization and Bundling of Actin Filaments

Actin exists as monomers (G-actin) and filamentous polymers (F-actin) and the maintenance of the balance between G-actin vs. F-actin is important for physiological functions including cell locomotion, cytokinesis, maintenance of cell shape and muscle contraction (Stricker et al., 2010). Two important actors control the polymerization and depolymerization of the actin filaments in cells: the ARP2/3 complex and cofilin. They work synergistically in such a way that the newly polymerized filaments from cofilin-generated barbed ends are ATP-rich filaments that promote the nucleation and branching activity of the ARP2/3 complex (DesMarais et al., 2004). Biochemical studies have shown that LUZP1 is an actin cross-linking protein (Wang and Nakamura, 2019a). As it has been proposed for EPLIN (a LUZP1 interactor) (Song et al., 2002; Maul et al., 2003), one hypothesis is that LUZP1 and EPLIN could regulate actin polymerization by influencing both assembly (nucleation, especially of ARP2/3 branched structures) and disassembly (stability) of F-actin, but this awaits detailed biochemical studies. We observed a reduction in ACTR3 in Luzp1^–/–^ cells compared to WT cells, suggesting that nucleation of branched actin might be diminished. In contrast, we observed an increase in phosphorylated (or inactive) cofilin levels, suggesting that Rho GTPase signaling might be activated upon loss of *Luzp1*. While normally this leads to increased stress fibers (Ridley and Hall, 1992), if the actin filaments are unable to become bundled due to absence of *Luzp1*, then phalloidin-positive stress fibers will be reduced, as we observed in Luzp1^–/–^ cells. As suggested by others (Liu et al., 2007), the elevated cofilin phosphorylation levels in Luzp1^–/–^ cells may reflect a compensatory response to weakened stress fibers.

### *LUZP1* Exhibits Frequent Genomic Aberrations in Cancer

In this work, we demonstrate that heterozygous loss of *LUZP1* is frequent in different cancer types. As observed in Luzp1^–/–^ cells, a switch from proliferation to migration/invasion is a common event in the context of cancer (Mehlen and Puisieux, 2006). However, the increase in apoptosis was puzzling. One possibility is that the cytoskeletal alterations and the multiple centrioles could lead to genomic instability. This phenomenon would be deleterious in benign cells, but tolerated in cancer cells. The heterozygous loss of *LUZP1* observed in tumor samples could be the consequence of the balance between the advantage of decreasing LUZP1 levels enough to promote invasion and the counterselection of complete loss to avoid genomic instability or cell division defects leading to apoptosis. Another perspective would be that complete *LUZP1* loss might be counterselected in cancer due to the antiproliferative and proapoptotic effects, and this explains the frequency of heterozygous losses.

In summary, our study demonstrates that LUZP1 controls proliferative and invasive features in cancer, thus providing a feasible explanation for its frequent copy number aberrations in various cancer types.

## Data Availability Statement

The raw data supporting the conclusions of this article will be made available by the authors, without undue reservation.

## Author Contributions

LB-B, JS, and RB designed the experiments, analyzed the data, and wrote the manuscript. LB-B, MG-S, VM, AE, NM-M, and JS developed the experimental protocols, performed the experiments, and analyzed the data. AC and JR provided the scientific resources. All authors contributed to the article and approved the submitted version.

## Conflict of Interest

The authors declare that the research was conducted in the absence of any commercial or financial relationships that could be construed as a potential conflict of interest.

## Footnotes

1 http://crispr.mit.edu/

2 http://portals.broadinstitute.org/tcga/home

## Supplementary Material

Supplementary Figure 1 LUZP1 knockout cells display more filopodia and more apoptosis than control. **(A)** Micrographs of representative individual WT and Luzp1^–/–^ cells used for Figure 2F. Filopodia were detected by WGA (magenta), F-actin by phalloidin (green) and nuclei by DAPI (blue). Yellow arrowheads point at regions containing filopodia. Black and white images show the single green and magenta channels. Scale bar, 2.5 μm. **(B)** Representative profiles of FACS analysis of apoptosis in WT and Luzp1^–/–^ cells analyzed in Figure 3C. Q2 and Q4 point at early and late apoptosis cells (blue and purple dots, respectively).
