# Supplementary figures and images for: LUZP1 Controls Cell Division, Migration and Invasion Through Regulation of the Actin Cytoskeleton
Source: Front Cell Dev Biol. 2021 Apr 1;9:624089. doi: 10.3389/fcell.2021.624089 (PMC8049182; doi:10.3389/fcell.2021.624089)

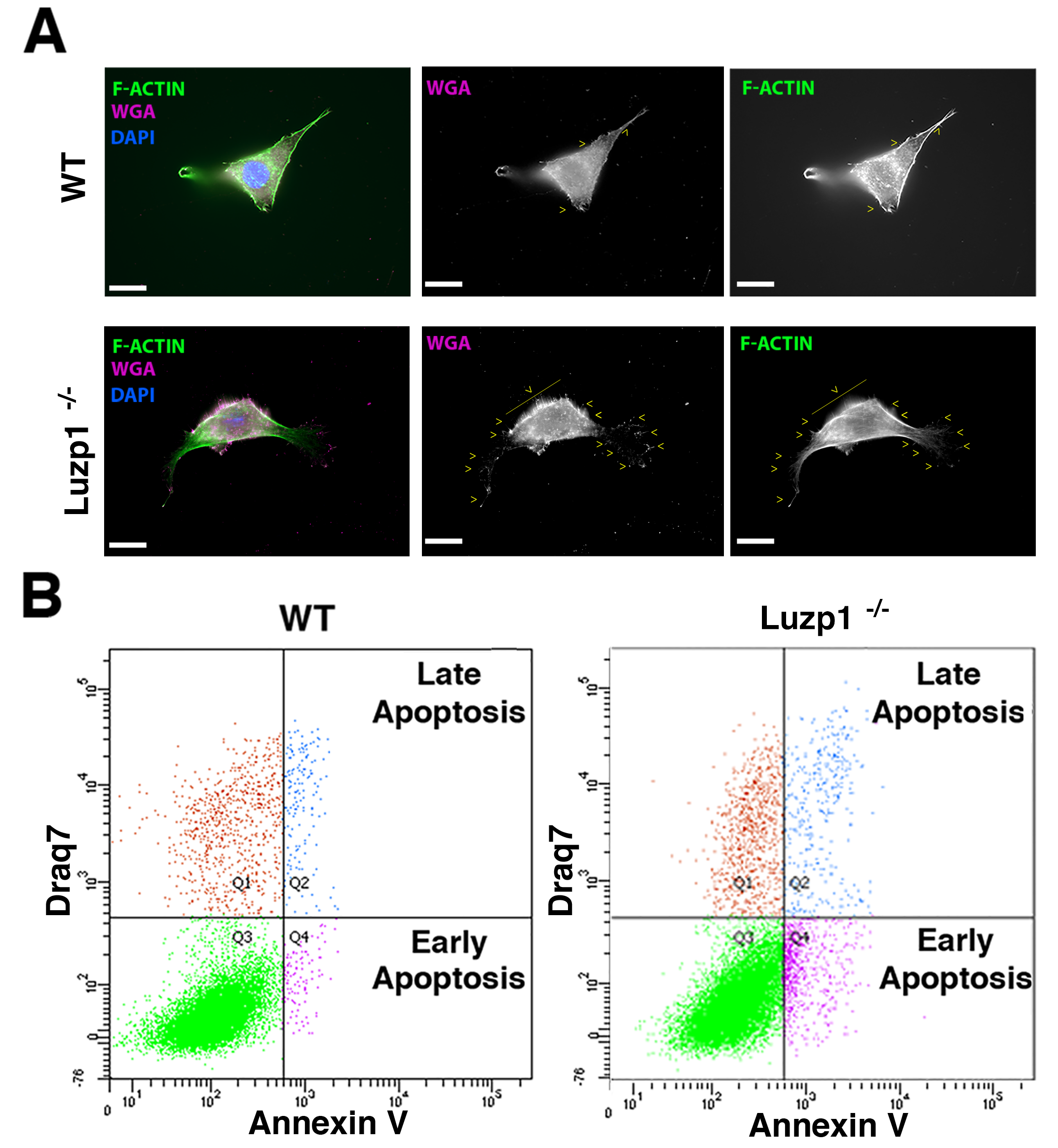

Supplement: Supplementary Figure 1 — LUZP1 knockout cells display more filopodia and more apoptosis than control. (A) Micrographs of representative individual WT and Luzp1–/– cells used for Figure 2F. Filopodia were detected by WGA (magenta), F-actin by phalloidin (green) and nuclei by DAPI (blue). Yellow arrowheads point at regions containing filopodia. Black and white images show the single green and magenta channels. Scale bar, 2.5 μm. (B) Representative profiles of FACS analysis of apoptosis in WT and Luzp1–/– cells analyzed in Figure 3C. Q2 and Q4 point at early and late apoptosis cells (blue and purple dots, respectively). [file Image_1.TIF]
